# Comparison of significant single nucleotide polymorphisms selections in GWAS for complex traits

**DOI:** 10.1007/s13353-015-0305-6

**Published:** 2015-08-21

**Authors:** M. Frąszczak, J. Szyda

**Affiliations:** Biostatistics Group, Department of Genetics, Wroclaw University of Environmental and Life Sciences, Kożuchowska 7, 51-631 Wroclaw, Poland; National Research Institute of Animal Production, Krakowska 1 A, 32-083 Balice, Poland

**Keywords:** Complex traits, GWAS, Mixed model, Significance testing

## Abstract

The goal of this study was to compare significant SNP selection approaches in the context of complex traits based on SNP estimates obtained by models: a model fitting a single SNP (M1), a model fitting a single SNP and a random polygenic effect (M2), the nonparametric CAR score (M3), a SNP-BLUP model with random effects of all SNPs fitted simultaneously (M4). There were 46,267 SNPs tested in a population of 2601 Holstein Friesian bulls, four traits (milk and fat yields, somatic cell score, non-return rate for heifers) were considered. The numbers of SNPs selected as significant differed among models. M1 selected a very large number of SNPs, except for a NRH in which no SNPs were significant. M2 and M3 both selected similar and low number of SNPs for each trait. M4 selected more SNPs than M2 and M3. Considering linkage disequilibrium between SNPs, for MY M2 and M3 selected SNPs more highly correlated with each other than in the case of M4, while for FY M3 selection contained more correlated SNPs than M2 and M4. In conclusion, if the research interest is to identify SNPs not only with strong, but also with moderate effects on a complex trait a multiple–SNP model is recommended. Such models are capable of accounting for at least a part of linkage disequilibrium between SNPs through the design matrix of SNP effects. Functional annotation of SNPs significant in M4 reveals good correspondence between selected polymorphisms and functional information as well as with QTL mapping results.

## Introduction

For many years genome-wide association studies (GWAS) have been a useful tool for detecting genetic variants associated with traits in human genetics (Visscher et al. 2012). With the advancement of genotyping technology high-density single nucleotide polymorphism (SNP) platforms have also been developed for other species including livestock. A whole bunch of statistical models have been applied to perform GWAS based on SNP chips. These vary from models where each SNP is considered individually to models with effects of all the available SNPs fitted simultaneously. However, the major drawback of interpreting GWAS results is the optimal selection of polymorphisms which are to be claimed as significantly associated with the analyzed trait and poor repeatability of results across methods and data sets. This problem is especially important when analyzing traits with a complex mode of inheritance (summarized by Visscher et al. 2012).

The variety of GWAS models applied to complex traits covers linear regression, penalized regression approaches with various shrinkage priors for SNP effects, like the least absolute shrinkage and selection operator regression (LASSO) introduced by Tibshirani (1996) (e.g., applied by Wu et al. 2009), the elastic net introduced by Zou and Hastie (2005) (e.g., applied by Do et al. 2011), ridge regression introduced by Hoerl and Kennard (1970) (e.g., applied and extended by Zhan and Xu 2012), normal exponential gamma distribution proposed by Hoggart et al. (2008), or by incorporating the functional information into SNP selection through biological pathways (Braun and Buetow 2011).

Our study was focused on the comparison of significant SNP selection approaches in the context of complex traits. Significance of SNPs selected based on estimates obtained from a mixed model routinely used in genomic evaluation of dairy cattle in Poland was compared to three single SNP models. In particular, the genomic selection model, which considers all polymorphisms simultaneously by applying shrinkage on SNP estimates through fitting a normal distribution with a predefined variance, is compared with (i) a linear model with a fixed effect of a single SNP which accounts neither for SNP intercorrelation, nor for family relationships (ii) a linear mixed model with a fixed, single SNP effect and a random additive polygenic effect for capturing the family relationships, and (iii) the CAR score regression proposed by Zuber and Strimmer (2011), which represents a fully nonparametric approach. Generally, we were interested in quantifying the differences between models in defining significant SNPs. Our particular focus though was on assessing the validity of the genomic selection model for GWAS purposes, since such a model is routinely evaluated on large and very informative data sets in many countries and, besides selection purposes, could be a potential source of information on single gene effects on traits undergoing selection.

## Materials and methods

### Data set

The material of our study consists of 2601 Polish Holstein-Friesian bulls genotyped with the Illumina BovineSNP50 BeadChip, which consists of 54,001 SNPs (version 1) and 54,609 SNPs (version 2). The applied SNP selection criteria comprised polymorphism, expressed by the minor allele frequency (MAF), with the minimum MAF of 0.01, and technical quality of a SNP expressed by the minimum call rate of 90 % within the analyzed sample of bulls. After quality control 46,267 SNPs were selected for further analysis.

Four traits undergoing a complex mode of inheritance were considered in the study: somatic cell score (SCS) representing a trait with “pure” polygenic mode of inheritance, milk (MY) and fat (FY) yields representing traits with a polygenic mode of inheritance enhanced by single genes with large effects, and non-return rate for heifers (NRH) as a trait with a very strong environmental component expressed by heritability of 0.02. Deregressed proofs of bulls were used as pseudophenotypes. Deregression, which was performed in order to remove ancestral information from the conventional breeding values of bulls, was based on a method proposed by Jairath et al. (1998). The corresponding conventional breeding values were estimated based on a random regression test day model for MY, FY, and SCS (Strabel and Jamrozik 2006) and based on a lactation model for NRH (Jagusiak and Żarnecki 2006) using phenotypic information corresponding to the routing national evaluation from April 2012. For each trait 2588 (SCS), 2601 (MY and FY), and 2524 (NRH) records were available.

### Models for SNP effect estimation

The SNP effects were estimated by four different models. Single SNP models comprising (M1) **y** = **μ** + **Xβ** + **ε** and (M2) **y** = **μ** + **Xβ** + **Z**_1_**α** + **ε** were solved using the ASReml3 software (Gilmour et al. 2009). In the above models **y** represents a vector of deregressed breeding values for MY, FY, SCS or NRH; **β** denotes a fixed SNP effect with a design matrix **X** ∈ {−1, 0, 1} for a homozygous, a heterozygous, and an alternative homozygous SNP genotype respectively; **α** denotes a random polygenic effect of a bull with an incidence matrix **Z**_1_, where **α** is distributed as $$ N\left(0,\mathbf{A}{\widehat{\sigma}}_{\alpha}^2\right) $$, with **A** being a relationship matrix for bulls and $$ {\widehat{\sigma}}_{\alpha}^2 $$ representing the estimate of total additive genetic variance of a given trait calculated elsewhere for the whole active population of Polish Holstein-Friesian dairy cattle. The residual effects vector **ε** is distributed as *N*(0, **D***σ*_*ε*_^2^) where **D** is a diagonal matrix weighted by the effective daughter contribution for each bull and *σ*_*ε*_^2^ denotes a residual variance; **μ** denotes an overall mean. The CAR score regression (M3) proposed by Zuber and Strimmer (2011) was selected as the third model for its simplicity and computational efficiency of variable ranking in linear regression based on the Mahalanobis-decorrelation of the explanatory variables representing a nonparametric approach. According to Zuber and Strimmer (2011) this approach is very effective computationally and yields prediction errors as well as true and false positive rates that compare favorably with other regression techniques such as elastic net and boosting. The CAR scores ω_i_, which were considered as the SNP selection criterion, are defined as: $$ \boldsymbol{\upomega} ={\mathbf{P}}^{-\raisebox{1ex}{$1$}\!\left/ \!\raisebox{-1ex}{$2$}\right.}{\mathbf{P}}_{\beta y} $$, where **P**_*βy*_ is the marginal correlation vector between deregressed breeding values and SNPs, **P** denotes shrinkage estimator given by: *λ****I***_***d***_ + (1 − *λ*)***R***_*empirical*,_ where $$ \lambda $$ is a shrinkage intensity and ***R***_*empirical*_ is the empirical non-regularized correlation matrix among SNP genotypes. The CAR criterion was computed using an R package CARE. The genomic selection model (M4) **y** = **μ** + **Z**_2_**g** + **ε**, equivalent to the so called SNP-BLUP model, is routinely used for the prediction of direct genomic breeding values for the Polish Holstein-Friesian population (Szyda et al. 2011). Here **Z**_2_ is a design matrix for SNP genotypes, which is parameterized as −1, 0, or 1 for a homozygous, a heterozygous, and an alternative homozygous genotype respectively and **g** is a vector of random additive SNP effects distributed defined as: $$ N\left(0,\mathbf{I}\frac{{\widehat{\sigma}}_a^2}{46267}\right) $$, with **I** being an identity matrix.

The SNP effects in M1 were estimated by weighted least squares with the effective number of daughters corresponding to teach observation y used as a weighting variable. To estimate **β** in M2 the objective function log *f*_*y*_(**y**|**α**, **β**, **G**) + log *f*_*α*_(**α**, **G**), where $\mathbf{G}=\mathbf{A}{\widehat{\sigma}}_{\alpha}^2$, was used. Differentiating it with respect to **β** and **α** leads to the mixed model equations (Henderson 1984). M3 is a model free procedure in which CAR scores are functions of empirical SNP-pseudophenotype and SNP-SNP correlations. The estimation of parameters of M4 was based on solving the corresponding mixed model equations using the iteration on data technique applying the Gauss-Seidel algorithm with residuals update (Legarra and Misztal 2008).

### Significant SNP selection

In case of M1, M2, and M4 the Wald test was used to obtain the nominal type I error corresponding to a standard normal distribution. For single SNP models M1 and M2 the resulting P values were subjected to multiple testing correction for the number of SNP tested (*N* = 46,267) via Bonferroni’s approach, while for a multiple SNP model M4 a nominal P value was used as a selection criterion. In M3 the null distribution of the function of CAR scores $t=\omega \sqrt{\frac{1-\kappa }{1-{\omega}^2}}$ was used for obtaining type I error rates, which follows the Student t distribution with (*κ* − 1) degrees of freedom estimated by the R package *fdrtool* (Strimmer 2008). For all models SNPs were selected as significant when P values associated with their estimates did not exceed the 0.001 threshold.

Genomic annotation of SNPs was performed using SNPchiMp (Nicolazzi et al. 2014) for the identification of SNP positions corresponding to the UMD3.1 bovine genome assembly and Variant Effect Predictor (McLaren et al. 2010) for the identification of genomic positions of SNPs.

## Results

### The number of significant SNPs selected

The numbers of SNPs selected as significant by different models are presented in Table 1. For MY, FY, and SCS the simplest model M1 always selected a very large number of SNPs ranging between 2242 (SCS) and 3398 (MY), widely exceeding the number of SNPs selected by M2-M4. Although models 2 and 3 markedly differ in modeling and hypothesis testing assumptions, they both select a very similar and low number of SNPs for each trait. The genomic selection model M4 is intermediate in terms of the number of SNPs selected.

**Table 1:** The numbers of SNPs selected as significant

Trait	M1	M2	M3	M4
Fat yield	2435	72	48	182
Milk yield	3398	66	78	153
Somatic cell score	2242	4	0	163
Non-return rate for heifers	0	0	0	125

Except for M4, traits with different putative inheritance modes resulted in different numbers of significant SNPs. For MY and FY the largest numbers of SNPs were selected at BTA14 — a chromosome harboring DGAT1 gene of high effect on milk production traits. For SCS, a trait with a pure polygenic mode of inheritance, a lower number of SNPs was indicated as significant, with only four SNPs selected by M2. For NRH, a trait with a very weak genetic component (as compared to the environmental based variation) no SNPs were identified by M1-M3. A very different pattern was observed for M4. Since all polymorphisms’ effects are estimated simultaneously with the underlying normal distribution shrinkage, the SNP selection procedure based on the 0.001 threshold chooses 0.1 % of the most significant SNPs regardless of a trait. As a consequence a very similar number of polymorphisms varying between 125 (NRH) and 182 (FY) for each trait was selected. Since, based on the very large number of SNPs selected, M1 does not seem to be a valid model for GWAS on complex traits, in further result description and discussion we confine ourselves to M2-M4.

### Influence of SNP informativeness on SNP selection

No marked differences between models were observed regarding the information content, expressed by MAF, between SNPs selected as significant by M2–M4. The average MAF of polymorphisms selected for MY by M2 amounted to 0.39, by M3 to 0.30 and M4 to 0.36 and was even more similar for FY with 0.37 (M2), 0.38 (M3) and 0.49 (M4). The average MAF in group of SNPs common between all considering models is 0.40 for MY and 0.38 for FY. On the other hand, considering MY, on average M2 and M3 selected SNPs which were more highly correlated with each other than was the case for M4 since the average LD expressed by pairwise correlation between significant SNPs was equal to 0.18 and 0.17 for M2 and M3, but only 0.08 for the genomic selection model. For FY, the average LD of 0.11 among SNPs selected by M4 was also lower from the corresponding values form M2 and M3 which were equal to 0.17 and 0.21 respectively. The highest average pairwise correlation was observed between significant SNPs which were common for models M2 and M3.

### Comparison of significant SNP sets between models

**Fig. 1:**
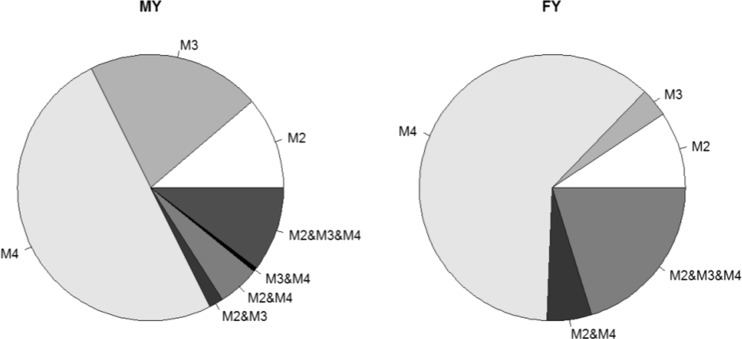
The percentage of SNPs significant between models

**Fig. 2:**
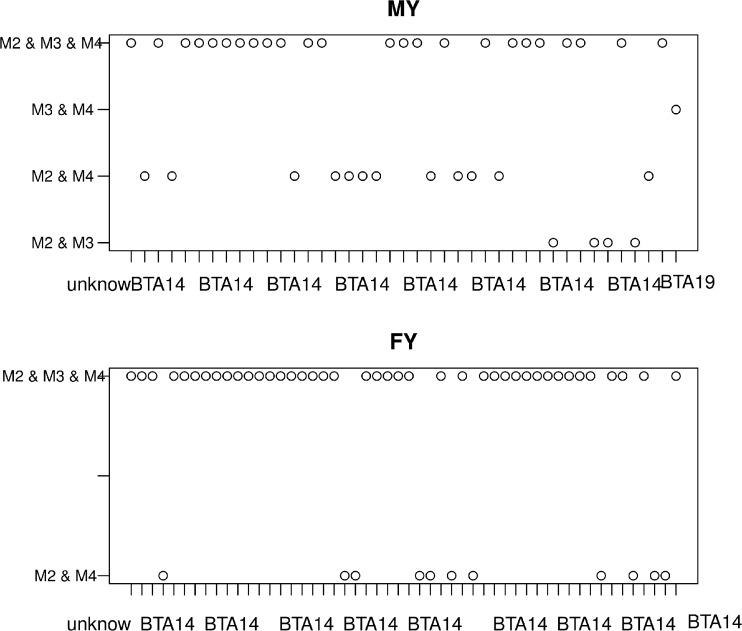
Genomic location of significant SNPs common between models

Figure 1 presents the percentage of SNPs significant between M2 and M4. For production traits a relatively large proportion of significant SNPs was common among all three models making 24 SNPs common for MY and 41 SNPs common for FY, while no common for all models polymorphisms were identified for SCS and NRH. Focusing on the genomic selection model M4 as the reference it is evident from Fig. 2 that more mutual SNPs existed between M4 and M2 than between M4 and M3. In particular, in addition to the polymorphisms common to all three models, the genomic selection model had 12 and 11 additional SNPs in common with M2 for MY and FY respectively, but only one SNP for MY mutual with M3. For MY there are four polymorphisms common for models M2 and M3.

### Genomic SNP annotation

**Fig. 3:**
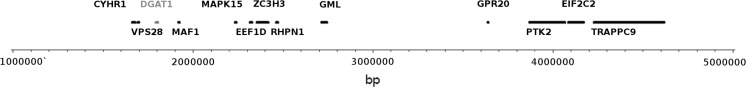
Position of genes on BTA14 marked by significant SNPs common for models 2–4

Genomic annotation was carried out for SNPs which were selected as significant by all three modles, i.e., M2, M3, and M3 (Table 2). Since M3 indicated no significant polymorphisms for SCS and NRH for those traits no annotation was considered. For FY 22 genes were marked by significant SNPs common between models, while for MY a lower number of eight genes was identified with six of them also significant for FY. All genes are located on BTA14 with varying distances from DGAT1 — a well known candidate gene for milk production traits (Grisart et al. 2002), ranging between 104,646 and 3,768,523 bp (calculated as a distance between corresponding significant SNP positions) (Fig. 3). Most of the SNPs are located within genomic range of the gene, mainly within intronic regions, but rs110323635 and rs41256919 lie in exons of MAPK15 and MAF1 genes respectively.

**Table 2:** Annotation of SNPs significant in M2, M3, and M4. Genes significant for both MY and FY are marked in bold. For significant genes all SNPs located within intron/exon regions are listed

Gene acronym	Gene name	Ensemble ID	BTA	SNP	SNP annotation	SNP position
Fat yield						
FOXH1	Forkhead box H1	ENSBTAG00000004761	14	rs109146371	3390 bp upstream	1,651,311
CYHR1	Cysteine/histidine-rich 1	ENSBTAG00000035254	14	rs109968515	intron	1,675,278
VPS28	Homologous to vacuolar protein sorting 28	ENSBTAG00000026320	14	rs17870736	intron	1,696,470
DGAT1	Diacylglycerol O-acyltransferase 1	ENSBTAG00000026356	14	rs109421300	intron	1,801,116
MAF1	Homologous to RNA polymerase III-inhibiting protein	ENSBTAG00000012242	14	rs41256919	exon – missense variant	1,923,292
SPATC1	Spermatogenesis and centriole associated 1	ENSBTAG00000026350	14	rs41629750	4472 bp upstream	2,002,873
PLEC	Plectin	ENSBTAG00000011922	14	rs109350371	460 bo upstream	2,054,457
**MAPK15**	Mitogen-activated protein kinase 15	ENSBTAG00000019864	14	rs110323635	exon – missense variant	2,239,085
EEF1D	Elongation factor 1-delta	ENSBTAG00000014643	14	rs109661298	intron	2,319,504
ZC3H3	Zinc finger CCCH-type containing 3	ENSBTAG00000021472	14	rs109617015	intron	2,386,688
RHPN1	Rhophilin, Rho GTPase binding protein 1	ENSBTAG00000002104	14	rs109529219	intron	2,468,020
	Novel gene	ENSBTAG00000003606	14	rs110199901	intron	2,524,432
**LY6K**	Lymphocyte antigen 6 complex, locus K	ENSBTAG00000000158	14	rs110174651	297 bp downstream	2,754,909
LY6D	Lymphocyte antigen 6 complex, locus D	ENSBTAG00000034498	14	rs110237430	978 bp downstream	2,803,998
LYPD2	LY6/PLAUR domain containing 2	ENSBTAG00000016210	14	rs109476486	2653 bp upstream	2,826,632
BAI1	Brain-specific angiogenesis inhibitor 1	ENSBTAG00000006385	14	rs109545018	intron	3,006,509
**GPR20**	G protein-coupled receptor 20	ENSBTAG00000015985	14	rs110411273	71 bp downstream	3,640,788
TSNARE1	t-SNARE domain containing 1	ENSBTAG00000009974	14	rs109875744 rs110888717 rs109295487	intron intron intron	3,078,843 3,117,493 3,137,184
**PTK2**	PTK2 protein tyrosine kinase 2	ENSBTAG00000009578	14	rs109670279 rs41624797 rs109131748 rs110185345	intron intron intron intron	3,885,798 3,956,956 4,017,201 4,043,743
**EIF2C2**	Argonaute RISC catalytic component 2	ENSBTAG00000001579	14	rs109948273 rs41576704 rs108980964	intron intron intron	4,103,850 4,127,413 4,149,375
**TRAPPC9**	Trafficking protein particle complex 9	ENSBTAG00000013955	14	rs109807697 rs109248069 rs111018678 rs110017379 rs55617160 rs110805364	intron intron intron intron intron intron	4,240,287 4,267,053 4,336,714 4,364,952 4,468,478 4,583,344
COL22A1	Collagen, type XXII, alpha 1	ENSBTAG00000015374	14	rs110444021 rs110351374	intron intron	5,225,467 5,428,037
Milk yield						
C8orf33	Chromosome 14 open reading frame 33	ENSBTAG00000000879	14	rs109752439	87 bp downstream	1,489,496
FAM135B	Family with sequence similarity 135, member B	ENSBTAG00000018218	14	rs110622450 rs109118650 rs110501942 rs109402117	intron intron intron intron	5,428,037 5,462,752 5,494,654 5,569,639

Although some of the genes may represent spurious associations arising through high LD to DGAT1, those which are located in more distant regions of BTA14 are potential candidate genes. Special attention is to be focused on EEF1D which according to a recent study of Xie et al. (2014) in dairy cattle exhibits significantly higher expression in mammary gland as in other tissues.

## Discussion

We observed that for complex traits approaches which model the genetic component by a single SNP — here represented by M1, which are very common and successful in GWAS for disease traits, have methodological problems. Since such models do not account for the polygenic background differentiating between SNPs in high LD, especially in the neighborhood of genes with strong effects is problematic and the multiple testing correction is not sufficient to remove the SNP effect upward bias due to correlated SNPs. An intensive post-processing of SNP estimates and/or corresponding P values regarding LD is then required.

A possible alternative, which is non-intensive computationally, is to use a single SNP model with the genetic background described by an additive polygenic effect (like M2) or a multiple–SNP without any assumptions regarding the inheritance mode (like M3). In our study M2 and M3 for MY and for FY, above and beyond the M2-M3, also the genomic model (M4) selected a similar number of SNPs as the number 55 resulting from the estimator proposed by Hayes and Goddard (2001) of the total number of genes influencing the variance of production traits (M): $M=N \ln \left(\frac{1-p}{p}\right)$, where *p* = 1/2*N*_*e*_. Note, that here we used the estimated number of heterozygous QTL segregating for production traits as *N* = 10.73 (Hayes and Goddard 2001) and the effective population size for dairy cattle as N_e_= 103 (Qanbari et al. 2010).

However, if the research interest is to identify SNPs not only with strong, but also with moderate effects on a complex trait a multiple–SNP model – here represented by M4, is recommended. Such models are capable of accounting for at least a part of LD between SNPs through the design matrix of SNP effects. A fine tuning of SNP selection procedure is needed based on the expected number of SNPs influencing the variability of a given trait, e.g., based on heritability estimates.

Recently, Wang et al. (2012) applied a genomic selection model (single step GBLUP) for GWAS on complex traits based on simulated data and found a good accuracy of prediction of QTL effects through SNPs. As indicated in that study it is important to realize that the selected SNPs do not necessarily represent underlying genes and that a chromosomal region in high LD (which is usually, but not always, equivalent to a region in physical neighborhood of the SNP) should be considered in search for causal mutations. Comparing the average LD of SNPs selected by single gene methods with the set selected by the genomic selection model it is evident that the latter is able to better deal with correlations between particular SNPs which occur through LD. Moreover, as pointed out by Dekkers (2012) a genomic selection model directly accounts for the population structure, not only on an averaged genome-wide level (through the additive polygenic covariance, as incorporated into M2), but also at a particular SNP sites (through the design matrix of SNP genotypes, as incorporated into M4). Another very important, practical advantage of using the genomic selection model for GWAS is that in most countries the model and underlying SNP effects are anyway evaluated on large, informative data sets.
